# An integrative ChIP-chip and gene expression profiling to model SMAD regulatory modules

**DOI:** 10.1186/1752-0509-3-73

**Published:** 2009-07-17

**Authors:** Huaxia Qin, Michael WY Chan, Sandya Liyanarachchi, Curtis Balch, Dustin Potter, Irene J Souriraj, Alfred SL Cheng, Francisco J Agosto-Perez, Elena V Nikonova, Pearlly S Yan, Huey-Jen Lin, Kenneth P Nephew, Joel H Saltz, Louise C Showe, Tim HM Huang, Ramana V Davuluri

**Affiliations:** 1 Human Cancer Genetics Program, Department of Molecular Virology, Immunology, and Medical Genetics, The Ohio State University, Columbus, OH 43210, USA; 2 Division of Medical Technology, School of Allied Medical Professions, The Ohio State University, Columbus, OH 43210, USA; 3 Department of Biomedical Informatics, The Ohio State University, Columbus, OH 43210, USA; 4 Medical Sciences, Indiana University School of Medicine, Bloomington, IN 47405, USA; 5 Department of Life Science and Institute of Molecular Biology, National Chung Cheng University, Min-Hsiung, Chia-Yi 621, Taiwan, Republic of China; 6 Institute of Digestive Disease, The Chinese University of Hong Kong, Hong Kong SAR, PR China; 7 Center for Systems and Computational Biology, Molecular and Cellular Oncogenesis Program, The Wistar Institute, Philadelphia, PA, USA

## Abstract

**Background:**

The TGF-β/SMAD pathway is part of a broader signaling network in which crosstalk between pathways occurs. While the molecular mechanisms of TGF-β/SMAD signaling pathway have been studied in detail, the global networks downstream of SMAD remain largely unknown. The regulatory effect of SMAD complex likely depends on transcriptional modules, in which the SMAD binding elements and partner transcription factor binding sites (SMAD modules) are present in specific context.

**Results:**

To address this question and develop a computational model for SMAD modules, we simultaneously performed chromatin immunoprecipitation followed by microarray analysis (ChIP-chip) and mRNA expression profiling to identify TGF-β/SMAD regulated and synchronously coexpressed gene sets in ovarian surface epithelium. Intersecting the ChIP-chip and gene expression data yielded 150 direct targets, of which 141 were grouped into 3 co-expressed gene sets (sustained up-regulated, transient up-regulated and down-regulated), based on their temporal changes in expression after TGF-β activation. We developed a data-mining method driven by the Random Forest algorithm to model SMAD transcriptional modules in the target sequences. The predicted SMAD modules contain SMAD binding element and up to 2 of 7 other transcription factor binding sites (E2F, P53, LEF1, ELK1, COUPTF, PAX4 and DR1).

**Conclusion:**

Together, the computational results further the understanding of the interactions between SMAD and other transcription factors at specific target promoters, and provide the basis for more targeted experimental verification of the co-regulatory modules.

## Background

SMAD transcription factors are the core members of transforming growth factor β (TGF-β) pathway, which has been implicated in the regulation of cell growth, differentiation, apoptosis and specification of developmental fate [1]. SMADs transmit signals from cell surface receptors to the nucleus in response to TGF-β. The general molecular mechanisms of the TGF-β/SMAD pathway from the cell membrane to the formation of a SMAD complex in the nucleus are fairly well established. Briefly, TGF-β elicits its molecular actions by binding to trans-membrane receptors, TGFBR1 and TGFBR2, which form an oligomeric complex and then transmit the signal into the cell via phosphorylation of SMAD2/3 proteins. Phosphorylated SMAD2/3 forms dimers or trimers with another protein, SMAD4, and this resultant SMAD complex is then translocated to the nucleus where it interacts with other DNA-binding co-regulators to modulate the transcription of TGF-β/SMAD target genes [1-3].

The TGF-β stimulated SMAD3/4 binds to 5'-GTCT-3', or its complement 5'-AGAC-3', called SMAD-BindingElement (SBE), with very low affinity [4]. It was initially thought that the presence of multiple SBEs in the target promoters likely enables tight binding, since activated SMAD complexes consist of SMAD oligomers. However, known SMAD target promoters seldom contain SBE concatemers, and those that contain up to four SBEs still require cooperating factors for effective DNA binding [5]. The list of DNA-binding SMAD partners, such as E2F1 [6], AP2 [7], PBX1 [8], OCT1 [9] and p300/CBP [10], is rapidly growing, and it is now believed that the high-affinity binding of the SMAD complex occurs through the incorporation of one or more different DNA-binding cofactors into the complex. Hence, the net effect of SMAD complex likely depends on transcriptional modules, in which the SBEs and partner transcription factor binding sites (TFBSs) are present in specific context. This mode of interaction provides a basis for high affinity and selectivity of target gene recognition and allows for the differential action of TGF-β in different cell types [11]. Thus understanding the complex nature of TGF-β/SMAD signaling requires knowing not only the set of genes bound and regulated by SMAD, but also its interacting transcription factors (together referred as SMAD modules) and the promoter regions where these interactions occur.

Abnormal activation or repression of TGF-β regulated processes is implicated in many diseases including renal, hepatic, and neurodegenerative disorders. Epithelial cells have a high turnover and their progenitor cells divide continuously, making them prime targets for genetic and epigenetic changes that lead to cell transformation and tumorigenesis [12]. In cancer development and progression, the TGF-β/SMAD signaling pathway functions as a double-edged sword, acting as a tumor suppressor in early tumorigenesis and as a tumor enhancer in late tumorigenesis [13]. While regulation of normal epithelial cell growth and differentiation is contingent upon appropriate up- or down-regulation of TGF-β/SMAD responsive genes, this homeostasis is disrupted during neoplastic processes, resulting in outgrowth and invasion of transformed cells. It has been reported that neoplastic cells become non-responsive to TGF-β/SMAD signaling activation, despite the fact that upstream regulators, such as TGFBR2, remain genetically intact [14-17]. It is suggested that other aberrant events, perhaps affecting co-regulators of this growth inhibitory pathway, trigger signaling perturbations in TGF-β/SMAD downstream targets. Although a few loci have been described in the literature, comprehensive identification of these co-regulator factors has yet to be performed [4,18-20].

In this study, we systematically identified TGF-β/SMAD regulated and synchronously coexpressed gene sets (defined as "synexpression" groups in [1]) on genome-scale by simultaneously conducting ChIP-chip (genome-wide location analysis of the chromatin) and mRNA expression profiling in an immortalized ovarian surface epithelial (IOSE) cell line. The identified target genes were classified into synexpression groups based on their temporal changes in expression after the TGF-β/SMAD signaling activation. Sequence analyses of target regions in each synexpression group revealed conserved SBEs and partner TFBSs. We applied the Random Forest (RF) [21] algorithm followed by Classification And Regression Tree (CART) [22] analysis to classify different synexpression target groups based on the presence of SBEs and binding sites of probable co-regulatory transcription factors. Several transcription factor modules were derived from this combined classification analysis, providing for the first time a comprehensive modeling of TGF-β/SMAD-co-regulator interactions in ovarian surface epithelial cells and important hypotheses for further experimental work.

## Results

### Identification of TGF-β/SMAD direct target promoters by ChIP-chip

Genome-wide discovery of TGF-β/SMAD targets was conducted by ChIP-chip using a promoter microarray of ~17,000 annotated promoter regions in the human genome. To probe the arrays, we performed different ChIP assays by using an antibody against SMAD4 in immortalized ovarian surface epithelial (IOSE) cells treated with TGF-β 1 for 0 and 3 hrs. The immunoprecipitated DNA was hybridized to an Agilent 44K promoter array (see Methods), and the experiments were repeated once [23]. We observed high positive correlation between the normalized log ratios (immonuprecipated DNA over total input DNA) of the biological replicate experiments, which demonstrates the reproducibility of the experiments (See Additional File 1 – Figure S1). A probe with weighted binding ratio at the 3-hr time point above 2 (*p*-value < 0.01) and having at least a 30% increase in relative binding compared to the 0 hr time point was considered as bound (see Methods).

### Identification of TGF-β/SMAD responsive genes by geneexpression profiling

In order to identify TGF-β transcriptionally responsive genes, we have used an Affymetrix U133 Plus 2 microarray to globally assess the gene expression in IOSE cells treated with TGF-β1 at 0, 3, 6, and 12 hrs. The experiments at each time point were repeated once, as described in Methods, and the gene expression estimates from the replicate experiments were remarkably similar (see Additional Files 2 and 3 – Figures S2 and S3 for reproducibility and clustering analyses of expression microarray). We performed Analysis of Variance to study the effect of time on gene expression and identify those genes that are differentially expressed in at least one time point (treated samples) as compared to the 0 hr control (untreated sample).

### Identification of synexpression groups of TGF-β/SMAD direct targets by intersection of ChIP-chip and gene expression data

**Figure 1:**
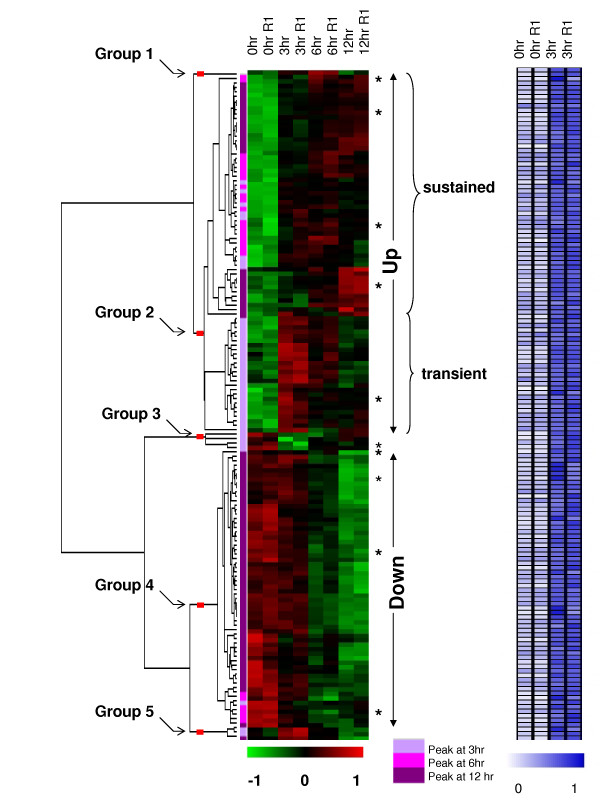
**Hierarchical cluster analysis of gene expression estimates from the expresssion microarrays and the heat map of binding ratios of the ChIP-chip experiments of the 150 TGF-β/SMAD target genes in IOSE cells**. Genes that showed both altered expression at 3, 6, or 12 hrs relative to 0 hr (*low-green and high-red*) and altered binding at 3 hrs compared to 0 hr (*low-white and high-blue*) after TGF-β signaling stimulation were shown. The expression data were median-centered and normalized to have a unit sum of squares for each gene before transforming to the color scale. Genes that were confirmed by ChIP-PCR and RT-PCR are indicated by asterisks. Results from the ANOVA analysis indicating the peak time point relative to 0 hr is indicated by a color bar. The experiments labeled R1 were biological replicates in the expression microarrays and were technical replicates in the ChIP-chip experiments. In the Chip-chip experiments, normalized binding ratios to have a unit sum of squares for each gene were used for the heatmap.

This analysis of ChIP-chip experiments identified 2,096 SMAD4-bound genes in IOSE cells, while expression microarrays identified 1,519 genes having expression changes following TGF-β treatment. By combining the results from the two microarray platforms, we identified 150 differentially expressed genes that were bound by SMAD4 in their promoter regions upon activation by TGF-β (See Additional File 4 – Table S1). One of the main goals of our study was to identify transcriptional modules containing SBE within the SMAD target promoters. Previous studies have demonstrated the utility of TFBS analyses of co-expressed gene sets to reveal *cis*-regulatory mechanisms in the target promoters [24,25]. We, therefore, performed hierarchical clustering of the expression data of the 150 genes (Figure 1) to determine different synexpression groups of TGF-β/SMAD direct targets. To identify informative gene clusters that correspond to major synexpression groups, we pruned the hierarchical tree and identified 5 branches, including two major (Groups 2 and 4) and three minor (Groups 1, 3 and 5) groups (See Additional Files 4 and 5 – Tables S1 and S2). We considered a group as major if it contained at least 10 genes. The major groups 2 and 4 respectively correspond to up- and down-regulatory expression patterns. Group 2 consists of 80 genes that showed elevated gene expression in at least one time point and group 4 consisted of 62 genes that showed decreased expression in comparison to 0 hr time point. Group 1, 3 and 5 had one, four and three genes, respectively. We, then, focused on the two largest groups, Group 2 and 4 for further analyses to derive the SMAD modules.

Target genes within the up-regulated group were further divided into two major and one minor branch. The expression level of the 54 genes in the major branch increased after TGF-β treatment and remained steadily high; we labeled this major branch the "sustained up-regulated group". On the other hand, the expression of the 25 genes in the middle branch had significantly increased at 3 hrs (*p*-values < 0.05 and fold increase > 1.5 in the comparison of 0 hr vs 3 hr), and returned to baseline expression over the 12-hr period. The genes in this synexpression group were labeled the "transient up-regulated group". The bottom branch had only one gene, *ATXN1*, with an elevated expression at the 3 and 12 hr time points and repressed expression at the 6 hr time point. Overall, we have identified 3 major synexpression groups – sustained up-regulated (54 genes), transient up-regulated (25 genes) and down-regulated (62 genes).

### Experimental validation of TGF-β/SMAD binding

**Figure 2:**
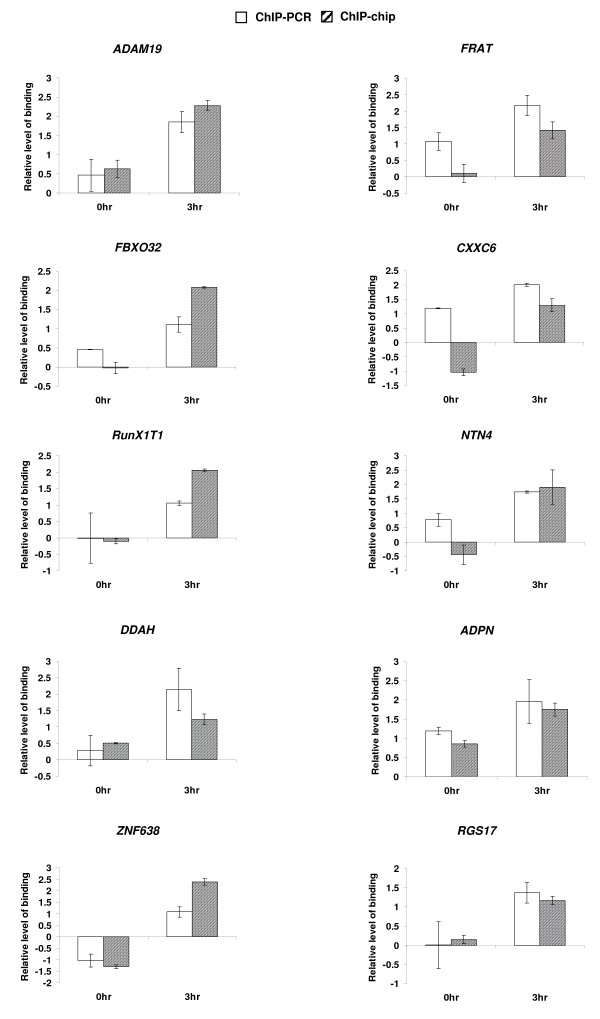
**Experimental validation of ChIP-chip results by ChIP-PCR analysis of 10 randomly selected TGF-β/SMAD target genes**. The cross-linked DNA from IOSE cells treated with TGF-β 1 of 10 genes were amplified by ChIP assays and measured by a real-time PCR machine. The ChIP-PCR values were from normalized experimental results from a standard curve, which was derived from total input DNA using the same primers. The plots are in log2 scale.

**Figure 3:**
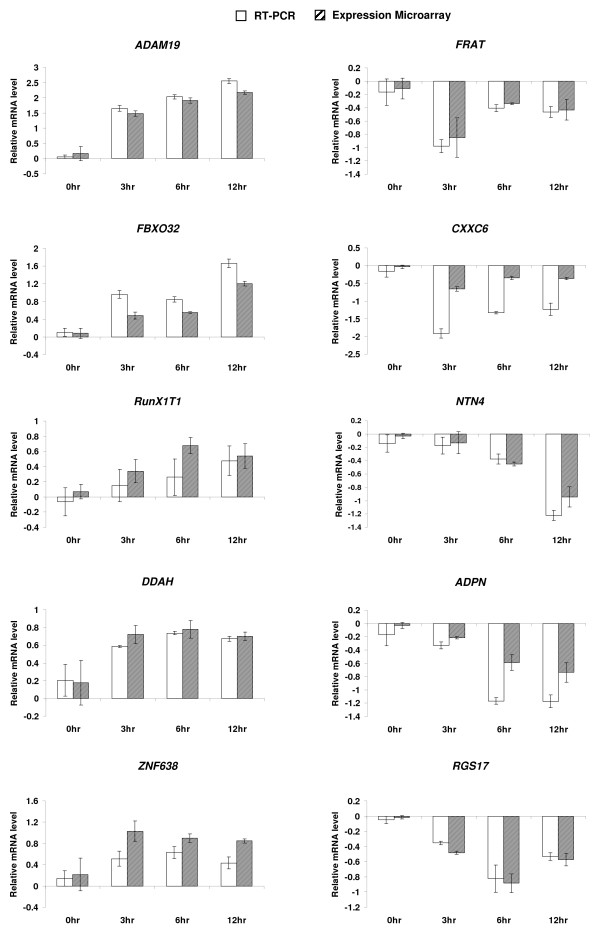
**Experimental validation of microarray results by RT-PCR analysis of 10 randomly selected TGF-β/SMAD target genes**. The mRNAs of 10 genes from IOSE cells, treated with TGF-β 1, were amplified by RT-PCR and measured by a real-time PCR machine. The fold-change in mRNA expression for each gene was calculated by setting the expression (RT-PCR or microarray) values at 0 hr to 1. The plots are in log2 scale.

SMAD4 binding of 10 randomly selected loci of the 150 targets that were shown to both bind SMAD4 and change gene expression in response to TGF-β, were confirmed in individual ChIP assays. On average, greater than 1.5 fold-enrichment was observed in IOSE cells after 3 hrs treatment with TGF-β1 (Figure 2). RT-PCR analysis was used to confirm altered expression of five group 2, two group 3, and three group 4 genes at 0, 3, 6 and 12 hrs after TGF-β stimulation (Figure 3). Specifically, we observed that the increase in expression of *ADAM19*, *FBXO32, RunX1T1*, and *DDAH *(group 2, sustained up-regulated) was maintained at the time-course between 3 and 12 hrs after treatment, while *ZNF638 *(group 2, transient up-regulated) showed increased expression at the 3 hr time point and gradual decrease to base-line level at the 12 hr time point. Decreased expression of *FRAT *and *CXXC6 *(group 3) was observed at 3 hrs after treatment. Expression levels of these two genes tended to increase afterwards, but remained below baseline levels by 12 hrs of treatment. On the other hand, expression of *NTN4, ADPN*, and *RGS17 *(group 4) continued to decrease at 6 hrs or 12 hrs after treatment. To summarize, the overall trends of temporal changes of expression and binding by SMAD4 observed in our microarray platforms were recapitulated by the RT-PCR and ChIP-PCR experiments, respectively.

### Random Forest variable selection followed by CART modeling identified novel co-regulatory modules in TGF-β/SMAD-responsive genes

In order to infer potential *cis*-regulatory SMAD modules and to discriminate target promoters of different synexpression gene groups, we applied classification methods driven by statistical learning approaches. For this classification analysis, we choose three major synexpression groups (sustained up-, transient up- and down-regulated from the hierarchical clustering) as different classes. The TFBSs, predicted by MATCH program, using the TRANSFAC (version 9.1) position weight matrices (PWMs), were used as the predictor variables of the classification function. Each predictor variable takes binary values 0 or 1 depending on the presence or absence of corresponding TFBS in the promoter region of interest. An attempt to classify the three synexpression groups (sustained up-, transient up- and down-regulated targets) by 3-class classifier resulted in very poor classification models (See Additional File 5 – Table S3), probably due to insufficient power as a result of the small sample sizes in each group. We, therefore, proceeded with a binary classification approach to build different classifiers for the two datasets – dataset 1 (up- vs. down-regulated targets) and dataset 2 (sustained up- vs. transient up-regulated targets).

**Figure 4:**
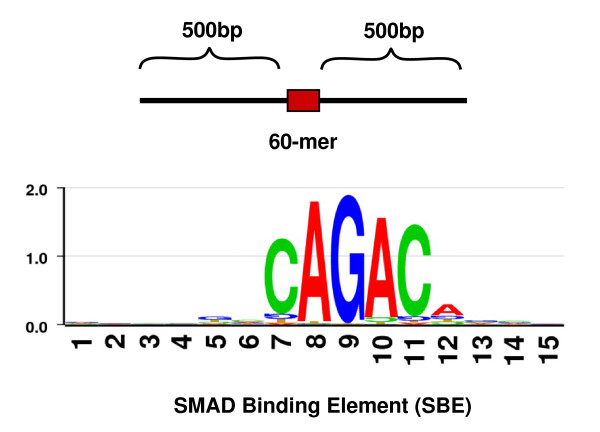
**Computational prediction of SMAD binding elements (SBEs)**. DNA sequences (1,060 bp) centered around each positive 60-mer probe sequence were used to detect SBEs. The degenerate nature of the binding site is shown by the SBE sequence logo, which was based on the 67 SBEs from literature (The logo was generated on ).

We determined the presence of SBEs in the 150 putative targets using the position weight matrix that we developed on 67 experimentally known SBEs. The consensus sequence of SBEs is highly degenerate with a 5-bp core sequence CAGAC (Figure 4). We scanned 1 Kbp region (500-bp on each side of the probe) around all the positive 60-mer probes on the promoter microarray and found that 124 (82.6%) of the aforementioned 150 loci contained at least one SBE (Figure 4, See Additional File 5 – Table S2). The criterion set for searching SBEs was based on the upper limit of sonicated DNA fragment length (i.e., ~500 bp) in the ChIP assay. To confirm the significant enrichment of SBEs in the target regions, we scanned for SBEs in a randomly selected 10,000 sequences of length 1,060 bp from the human genome. We found that 71% of these regions contained at least one SBE at the same cutoff used for scanning the target regions. This suggests that there is a significant enrichment of SBEs (Fisher's exact test *p*-value 0.001) in the 150 putative targets in comparison to the random set of sequences of similar length.

Next, we computationally analyzed the 124 loci for the presence of other TFBSs. A 440-bp sequence region centered on one SBE was obtained. This window (-220 bp, +220 bp) was used because 220 bp is the estimated length of DNA in one nucleosome. For sequences with more than one SBE, the one closest to the center of the 1,060 bp region was chosen. The MATCH program was used to predict the TFBSs [26]. These binding sites, present in at least 35% of either group (up- or down-regulated targets for dataset 1, sustained or transient targets for dataset 2), were retained in the data matrix as predictor variables. The 35% cut-off was arbitrarily chosen to keep the number of the predictor variables within a reasonable range. To increase our confidence, we tested and found that variation of this cut-off from 20% to 40% did not influence the outcome of the analyses. The final data-matrices contained a set of 164 and 159 TFBSs as predictor variables for dataset 1 and 2, respectively.

**Figure 5:**
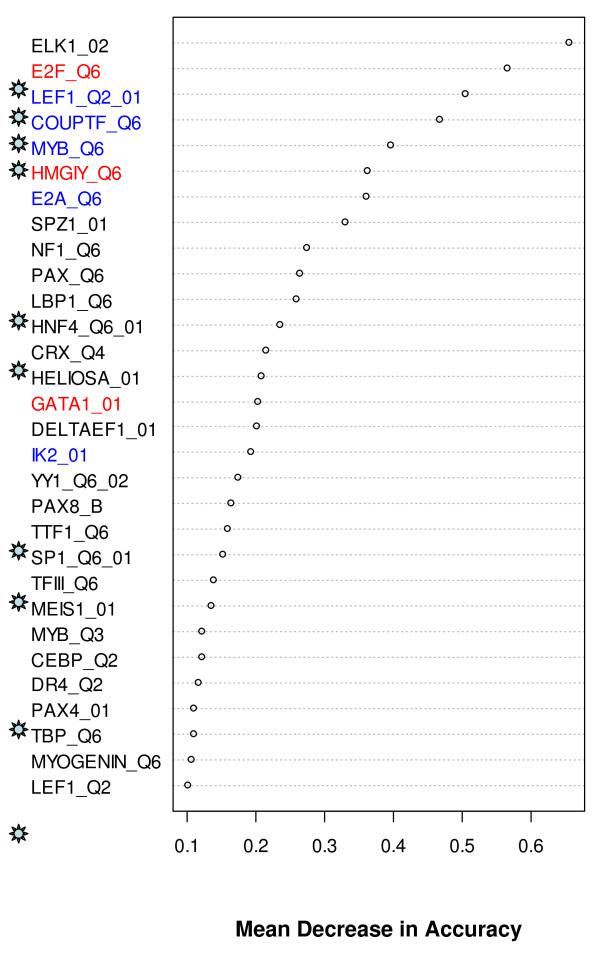
**Top 30 TFBSs selected by RF and their mean decrease in accuracy in up- vs. down-regulated SAMD target genes**. The mean decrease in accuracy was an average of 100 runs of RF. Experimentally known SMAD interacting TFBSs are marked with ☼. Statistically significant (*p*-value < 0.05 by Fisher's exact test) over-represented TFBSs are labeled in red and blue colors: red, over-represented in up- compared to down-regulated; Blue, over-represented in down- compared with up-regulated genes.

**Figure 6:**
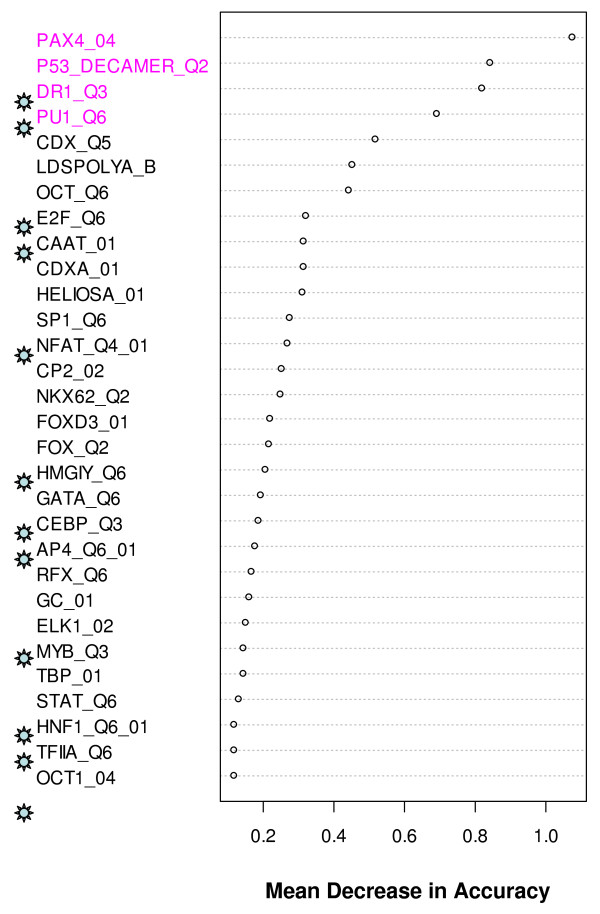
**Top 30 TFBSs selected by RF and their mean decrease in accuracy in sustained vs. transient SAMD target up-regulated genes**. The mean decrease in accuracy was an average of 100 runs of RF. Experimentally known SMAD interacting TFBSs are marked with ☼. Statistically significant (*p*-value < 0.05 by Fisher's exact test) over-represented TFBSs are labeled in magenta – over-represented in the sustained group as compared with the transient group.

We initially fitted CART and RF models to our data. A direct application of these models did not provide satisfactory prediction accuracies (Table 1). However, a feature of RF that is especially relevant in the current analyses is the variable importance measure, which estimates the relative importance of the TFBSs in discriminating one group from another and helps to select the TFBSs that are probably involved in the SMAD modules. RF provided a rank for each of the prediction variables based on mean decrease in accuracy of classification. We present the top 30 ranked variables identified by RF analyses in Figures 5 and 6. We also collected transcription factors that are known to interact synergistically with SMAD from published literature and the information is presented in Table 2. It is interesting to note that more than one third of the TFs in the RF generated lists (Figures 5 &6) are previously known to interact cooperatively with SMAD. On the other hand, of the 22 known SMAD co-regulators in Table 2, 27% and 45% were respectively represented in the top 30 TFBSs selected by RF from datasets 1 and 2 (See Additional File 5 – Table S4). We then fitted the CART model on subsets of these pre-selected variables. Using this implementation, the misclassification rates were dramatically improved in all the cases (Table 1). Although some misclassification rates were still high, the classification model and the RF generated lists provided an important first step in the direction of predicting cis-regulatory modules involving SMAD.

**Table 1:** Misclassification rates by CART and RF modeling

		Error rate	
	Number of Independent variables	Class 1	Class 2
**Dataset 1: Down/Up**		**Down**	**Up**
Sample Size		51	65
CART	164	0.41	0.46
RF	164	0.59	0.31
RF + CART	4	**0.37**	**0.23**
**Dataset 2: Transient/Sustained**		**Transient**	**Sustained**
Sample Size		23	41
CART	159	0.22	0.68
RF	159	0.86	0.19
RF + CART	3	**0.17**	**0.27**

For each dataset, the synexpression group labeling was the dependent variable and the TFBSs were the independent variables. CART model was derived by using Gini splitting criterion, equal prior setting, unitary cost and a 10-fold cross validation. The best tree was selected by minimum cost. The error rates were the rates on the test sample by cross validation. RF was run with stratified sampling with an equal sample size for both classes, whereas the sample size was set to the one of the class with smaller number of observations. The error rates were the average of out-of-bag error rates of 100 runs of RF, each with 1000 trees. RF + CART was to build a CART model on the top most important variables selected by RF. For both datasets, RF + CART provided the best classification results with lowest misclassification rates.

**Table 2:** List of transcription factors that are known to interact synergistically with SMAD (collected from literature search)

**Cofactors**	**Target genes**
GATA4	*IAP*, *IFABP *[74]; *INHA *[75]

C/EBPβ, E2F4/5, FoxO	*p15INK4b *[76]; *c-MYC, p21Cip1, GADD45A, GADD45B, IER1, CTGF, JAG1, LEMD3, SGK, CDC42EP3*, and *OVOL1 *[77]

STAT3	*GFAP; HP *[78-80]

LEF/TCF	*MYC *[81]; *Xtwn *[52]

TCF/β-catenin, AP1	*Gastrin *[82,83]

MYOCD	*SM22alpha, Tagln *[84]

P53	*AFP *[85], AFP [56]

SOX9	*COL2A1*[86]

COUPTF	*COL7A1*[54].

SP1, SP3	*LPL *[87]; *vimentin *[88]

ETS-1	*CCN2 *[89]

NFKappaB and AP1	*IL6 *[90]

HIF1α	*VEGF *[91]

AP-1	*ET1 *[92]

HNF-4	*apolipoprotein C-III *[58]

TFE-3	*SERPINE1 *(*PAI-1*) [93]

MITF	*mmcp-7 *[94]

OCT-1	*GATA2 *[95,9]

AP-2	*Col7a1*[7]

RARγ	human gene promoter construct [59]

**Figure 7:**
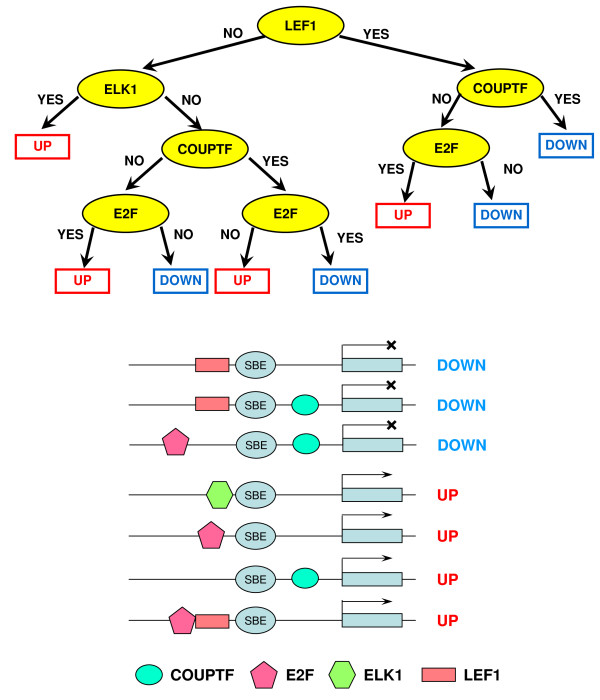
**Classification model that discriminates up- and down-regulated TGF-β/SMAD targets and corresponding SMAD co-regulatory modules**. Upper panel shows the CART model that discriminates between the TGF-β/SMAD up- and down-regulated targets. Lower panel shows the derived *cis*-regulatory modules of these target genes identified by the CART model. In the CART trees, "yes" means the TFBS was present and "no" means the TFBS was absent.

**Figure 8:**
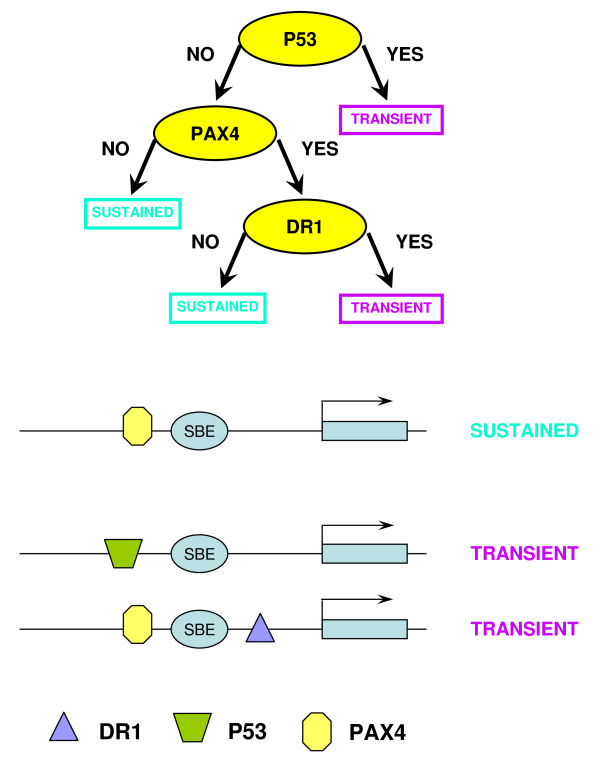
**Classification model that discriminates sustained and transient TGF-β/SMAD targets within the up-regulated group**. Upper panel shows the CART model and lower panel shows the derived *cis*-regulatory modules of these target genes identified by the CART model.

The binary trees constructed by CART are presented in Figures 7 and 8. Six TGF-β/SMAD co-regulators that could influence the expression status of target genes in each synexpression group, LEF1, ELK1, COUPTF, E2F, P53 and PAX4, were identified. The presence or absence of four of these TFBSs (LEF1, ELK1, COUPTF and E2F) distinguished between up- and down-regulated TGF-β/SMAD targets, and binding of P53 and PAX4 was associated with up-regulated genes (sustained and transient groups). The DR1 in the CART tree that discriminates the subgroups of up-regulated targets stands for Direct Repeat 1, a DNA site bound by transcription factors PPAR, HNF-4, COUPTF and RAR from the family of thyroid hormone receptor-like factors. Table S5 (See Additional File 6) contains the list of modules and their target genes. Interestingly, several up-regulated target genes (PTHLH, DKK1 and CFLAR), predicted to have a P53 binding site, have been validated experimentally by others, indicating that our prediction approach is correct for these three genes [27-29]. To further validate our model, we performed RNAi knock-down experiments for ADAM19 [30], a gene predicted to have SMAD4 and E2F binding sites and whose expression is upregulated in normal ovarian epithelial cells (Figure 3). However, only a slight decrease in ADAM19 expression was observed after knock-down of SMAD4 in TGF-β-treated IOSE, suggesting that additional factors regulate expression of this SMAD4 target gene [30]. In this regard, our future experiments will investigate a role for E2F play in the control of ADAM19 expression.

### Functional analyses of TGF-β/SMAD-responsive genes

**Figure 9:**
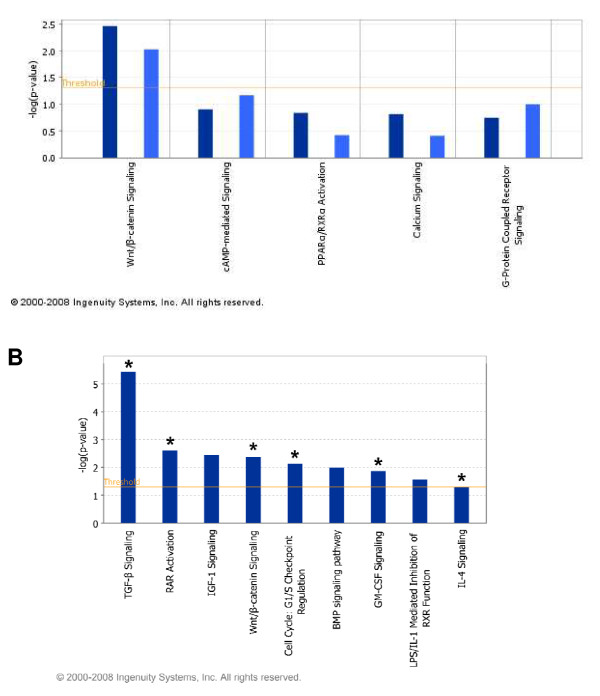
**Ingenuity Pathway Analysis of SMAD targets: (A)** A graphical representation of overlapping signaling pathways detected in differentially expressed genes and SMAD-target genes. Wnt/β-catenin was found to be significant. (**B**) The result of Ingenuity analyses of Affymerix gene expression and SMAD-predicted datasets. There are total nine significant signaling pathways (shown in bar graphs) for gene expression data, and six significant signaling pathways for SMAD-predicted molecules (shown by asterisks). There is a major functional overlap (about 70% or 6/9) between gene expression and SMAD-predicted data. Note that the p values were calculated by Ingenuity algorithm  per each pathway within Affymerix gene expression dataset and minus log p values are shown. The minus log p values for SMAD-predicted molecules are not shown.

We performed Ingenuity Pathway Analysis (Ingenuity^® ^Systems, , IPA 6.0) in order to find significant molecular functional categories in SAMD-target gene set and transform the target genes into a set of relevant networks by using literature-based records that are maintained in the Ingenuity Pathway Knowledge Base. We first performed IPA analyses independently on SMAD-responsive genes (from Affymetrix microarray analysis) and SMAD target genes (from ChIP-chip data analysis). The analyses produced 20 significant molecular and cellular functions (See Additional File 7 – Figure S4) that are significantly enriched in both gene sets. We found considerable overlap in the predicted pathways from both the data sets, and Wnt/β-catenin signaling pathway as one of the most significant pathways (Figure 9 and Table 3). Further, LEF1, an important transcription factor of β-catenin pathway, was predicted as a co-regulator of SMAD in our predicted SMAD modules. The cooperation between TGF-β and Wnt signaling pathways was also shown by several of earlier studies [31].

**Table 3:** Functional comparison of SMAD responsive (from gene expression) and SMAD-target (from ChIP-chip) gene sets obtained using Ingenuity Core analyses

**IPA Signaling Pathway**	Affymetrix (1095 genes)			SMAD predicted (150 targets)	
	
	IPA ratio	IPA p-value	No. molecules	IPA ratio	IPA molecules
TGF-beta signaling	0.169	3.68E-06	14	0.012	SMAD3

RAR activation	0.08	2.50E-03	15	0.011	GTF2H2, SMAD3

IGF-1 signaling	0.108	3.58E-03	10	n/a	n/a

Wnt/beta-catenin signaling	0.084	4.12E-03	14	0.018	CSNK1A1, DKK1, FRAT1

Cell cycle: G1/S checkpoint regulation	0.117	7.37E-03	7	0.033	HDAC9, SMAD3

BMP signaling	0.097	1.01E-02	10	n/a	n/a

GM-CSF signaling	0.113	1.34E-02	7	0.016	CAMK2D

LPS/IL-1 mediated inhibition of RXR function	0.065	2.68E-02	13	n/a	n/a

IL-4 signaling	0.1	5.15E-02	7	0.014	NFAT5

Only significant signaling pathways are listed. Ratio shows the relative overlap between number of molecules per pathway within each dataset over the total number of pathway-specific molecules present in Ingenuity Knowledge Base.

## Discussion

Through the interactions of different co-regulators, specific transcription factors can regulate different cellular processes which sometimes lead to opposite downstream effects [32,33]. SMAD transcription factors rely on transcription cofactors for appropriate activation or repression of target genes in response to TGF-β. TGF-β/SMAD signaling pathway is important for growth inhibition in normal ovarian epithelium, thus signaling disruption may lead to ovarian tumorigenesis [13]. Although primary ovarian cancer retains TGF-β-mediated growth inhibition, studies demonstrated that most ovarian cancer are non-responsive to TGF-β signaling pathway [15,34]. The cell line that we used in this study is responsive to TGF-β thus allowing us to evaluate the disruption of TGF-β signaling pathway in ovarian cancer. While most of the recent studies used expression microarrays to interrogate TGF-β targets in a particular system, those approaches cannot differentiate between direct and in-direct TGF-β/SMAD4 targets [35,36]. In our study, we combined both ChIP-chip and expression microarray data to identify TGF-β/SMAD4 targets. This approach provides important information regarding the direct TGF-β/SMAD4 targets as they relate to ovarian biology. The regulatory module that we identified may also be important in understanding the disruption of TGF-β signaling in ovarian cancer. Only 17 out of the 150 TGF-β/SMAD targets were previously known to respond to TGF-β in various systems (See Additional File 4 – Table S1). Thus, more than 90% of the targets identified in this study are novel targets.

To gain further insight into the potential biological relevance of these newly identified TGF-β/SMAD targets, we classified the 150 targets by biological functions. This analysis revealed that the majority of the targets are related to either signaling pathways or play a role in transcriptional regulation (See Additional File 7 – Figure S4). For example, expression of the kit ligand, KITLG, is downregulated after addition of TGF-β, a result consistent with a previous study demonstrating that treatment of rat ovarian surface epithelial cells with TGF-β results in KITLG downregulation [37]. Interestingly, KITLG is an important regulator of ovarian surface epithelial cell growth, and up-regulation of KITLG expression has been reported in ovarian cancer [38,39]. Taken together, these observations suggest that disruption of TGF-β signaling pathway may lead to altered KITLG expression, which in turn could contribute to ovarian cancer carcinogenesis. An exciting extension is the possibility of KITLG activation in ovarian cancer initiating cells (OCICs), as we recently reported upregulation of the KITLG receptor, c-kit/CD117, in this highly tumorigenic subpopulation of cells in human ovarian adenocarincomas [40].

It is interesting to note that TGFBR2 and SMAD3 were among the down-regulated targets, suggesting a possible negative feedback loop in the pathway. Since our microarray platform spans only the promoter regions, the number of binding sites in the present study is likely to be an underestimate at the whole genome level as recent finding indicates that the transcription factor binding happens throughout the genome [41]. On the other hand, we have identified a total of 1946 SMAD4 targets by ChIP-Chip, but only 150 shows expression changes. The other 1796 targets, however, may show expression changes after 12 hours of TGF-β stimulation but could not be detected in the current setting. Alternatively, those targets may require other transcription factor(s) for initiation. Although the presence of false positive cannot be excluded, our experimental design, in which the ChIP-chip data is subtracted from 3 hr after addition of TGF-β to 0 hr, should have minimized this possibility.

In order to decipher complex gene regulatory networks associated with signaling pathways that play critical roles in normal and aberrant cell behavior, accurate prediction of transcription factor co-regulatory modules is essential [42]. Computational analyses that rely solely on motifs derived from position weight matrix scanning are considered far from perfect and known to produce both false-positive and false-negative results [26]. Phylogenetic footprinting, can be used to identify conserved sequences between distantly related species thereby improving module discovery [43]. However, this comparative genomics approach can only partially improve prediction accuracy due to the lack of conserved binding sites among species and the unavailability of human gene counterparts in other organisms for comparative genomic analysis. With the advance of ChIP-chip technologies, we can now computationally interrogate the interactions between *cis*-acting elements and transcription factors using experimental data [44]. Recently, computational approaches that combine seemingly disparate experimental data have been successful in developing concise pathway models and transcriptional modules [45,46]. RFs have been receiving increased attention in the data-mining field as a means of variable selection in many classification tasks in computational biology, including the selection of a subset of genetic markers and genes in microarray data analysis relevant for the prediction of a certain disease [47-50]. Here, we have used an integrative modeling approach that combines CART and RF to classify different SMAD target promoters with reasonably good classification accuracy and reduced instability. Other popular classification methods, such as Naïve Bayes Tree, Logistic Model Tree, Bagging and LogitBoost (reviewed in [51]) or combination of these algorithms with RF may give different performance results and derive different SMAD modules, which needs a systematic testing. Although the main goal in classification is to build a model with minimal mis-classification error in cross-validation (Table 1), in this application we are equally interested in identifying TFBSs as highly important discriminating variables. One of the main goals of our analyses is to select potential SMAD interacting transcription factors from a large feature space (>150 transcription factors from Transfac database) in order to build SMAD modules. RF algorithm generates internal estimates of the decrease in the classifier's overall accuracy if that particular variable was not used in building the classifier. Thus, variables (TF binding sites) with larger importance measures can be deemed to have more power in discriminating different groups. A notable fact about our RF feature selection procedure is that more than one third of the transcription factors in the top ranking variables (Figures 5 and 6) are previously known to synergistically interact with SMAD in regulating the target promoter (Table 2). Conversely, a substantial number of the known SMAD co-regulators appeared as the most important variables (See Additional File 5 – Table S4). This demonstrates the power of RF feature selection procedure and indicates that other top ranking transcription factors could be novel partners of SMAD, resulting in different transcriptional outcomes.

We first built a large number of RFs to identify and rank TFBSs of importance; and then supplied the resultant TFBSs as a relatively smaller set of predictor variables to CART for classification, using step-wise forward selection procedure. Based on our original microarray data, this process dramatically improved the misclassification error rate compared to using CART or RF analysis alone. By running a large number of RFs, we obtained a stable rank for the most important variables, which could not be achieved with a single RF run. When fitting the CART model, a series of models were built, starting with the most important TFBS as the predictor variable, followed by systematically adding more TFBSs from the variable reservoir. As expected, the overall misclassification error rate (defined as the sum of the error rates for the individual groups) first decreased and then increased again (See Additional File 8 – Figure S5). The one at the bottom of the decreasing trend is the best model, overcoming the limitation of using pre-set arbitrary cutoff values for variable selection in other CART models [42].

By computational prediction, 83% of the target promoters contained SMAD4 consensus sequences, a significant enrichment compared to a random set of sequences. The consensus sequence of the SBE, nevertheless, contains only a weak signal. To ensure the binding of the SMAD complex, the presence of the binding sites of the co-regulators is equally important. Based on the ChIP-chip data, the combined classification tree analysis accurately predicted previously known TGF-β/SMAD co-regulators, including LEF1 [52], ELK1 [53], COUPTF [54], E2F [55], and P53 [56]. The transcription factors that recognize the DR1 site, PPAR, HNF-4, COUPTF and RAR are all known SMAD partners [54,57-59]. The combined RF and CART analysis also uncovered a novel co-regulator, PAX4, a paired-homeodomain transcription factor and important regulator of pancreas development [60,61]. Previous studies have demonstrated regulation of PAX4 expression by activin A, a TGF-β superfamily member, and transcriptional regulation via interactions between paired domain transcription factors PAX8 and PAX6 and SMAD [62,63]. Therefore, it seems reasonable to suggest that our computational prediction of a PAX4-SMAD interaction and subsequent target gene co-binding could contribute to gene up-regulation (Table S5). Furthermore, as a potential tumor suppressive function for PAX4 has recently been reported [64], we speculate that disruption of PAX4 could compromise TGF-β-mediated growth inhibition and contribute to ovarian carcinogenesis.

Our integrative computational modeling and Ingenuity Pathway Analysis suggests that SMAD and LEF1 co-regulate some of the up-regulated SMAD responsive genes. It was shown that the activation of MSX2 gene was mediated via the cooperative binding of SMAD4 at two SBEs and of LEF1 at two Lef1/TCF binding sites [65]. However, these predicted SMAD regulatory modules need to be confirmed by biological experiments. First, quantitative ChIP-PCR with the antibody against a TF can corroborate the recruitment of the TF to the promoter region of the target gene. Second, a promoter of a target gene with a TFBS deleted can be compared to a wild-type promoter to see if the TFBS confers any biological activity in a promoter-reporter assay setting. Third, comparison of target gene expression levels in cells transfected with siRNAs against specific SMAD proteins, or against a TF predicted to be a SMAD co-regulator, or against both, can reveal if there is any synergistic action between the two interacting partners.

## Conclusion

In conclusion, we have identified 124 TGF-β/SMAD targets by microarray experiments coupled with bioinformatics. Further computational analysis using CART and RF modeling has identified several transcription factor modules and provided important information in the transcriptional control of TGF-β/SMAD signaling. Guided by this computational information, experiments are underway to verify these co-regulator modules in normal and aberrant conditions such as ovarian cancer, a disease in which dysregulation of TGF-β/SMAD signaling has recently been demonstrated [66].

## Methods

### Chromatin Immunoprecipitation Microarray (ChIP-chip)

IOSE cells were maintained in a 1:1 mixture of medium 199 (Sigma, St. Louis, MO) and 105 (Sigma) supplemented with 10% FBS, 400 ng/ml hydrocortisone (Sigma), 10 ng/ml EGF and 50 units/ml of penicillin/streptomycin (Invitrogen, Carlsbad, CA). The cells were treated with 10 ng/ml of TGF-β1 (Sigma) for 3 hrs and then crosslinked with 1% formaldehyde for 10 min. Chromatin immunoprecipitation was performed by using a ChIP assay kit (Upstate Biotechnology, Lake Placid, NY) as described previously [42]. In brief, 1 × 10^6 ^cells were sonicated and immunoprecipitated by incubation with an anti-SMAD4 polyclonal antibody (H552, Santa Cruz Biotechnology, Santa Cruz, CA). Immunoprecipitated (ChIP) and total input DNAs were amplified by ligation-mediated-PCR for 24 cycles. Two μg of ChIP-DNA or input DNA were aminoallyl-labeled using the BioPrime DNA labeling system (Invitrogen) and then coupled with Cy5 and Cy3 fluorescence dyes (Amersham, Buckinghamshire, UK), respectively. DNAs were then co-hybridized to a modified version of whole genome oligonucleotide microarray (~44,000 60-mer probes; Agilent Technologies, Palo Alto, CA) representing the promoter regions of ~17,000 unique genes [67]. Following hybridization, the arrays were washed and then scanned using a GenePix 4000A Scanner (Axon, Union City, CA), followed by analysis with GenePix Pro 6.0 software (Axon).

### Expression Microarray

Total RNAs from IOSE cells treated with TGF-β1 at 0, 3, 6, and 12 hrs were extracted using TRIzol reagent (Invitrogen) following the manufacturer's instructions. In brief, about 1.5 μg of total RNA were reverse transcribed into cDNA using a HPLC-purified T7-oligo(dT) primer and Superscript II reverse transcriptase (Invitrogen). Biotinylated antisense cRNAs were then generated by *in vitro *transcription using the Bioarray RNA transcript labeling kit (Enzo Life Sciences, Farmingdale, NY). Fifteen μg of fragmented cRNAs were then hybridized to a Human Genome U133A plus 2.0 GeneChip Oligonucleotide Array (Affymetrix, Santa Clara, CA), representing over 47,000 transcripts in the human genome. Finally, the microarray slides were scanned using a GeneChip Scanner 3000 (Affymetrix) and analyzed by GeneChip Operating Software.

### Microarray Analysis

For ChIP-chip, low quality spots that were flagged by the software and spots that had a percentage of pixels with intensities more than two standard deviations above the background for each color ≤ 50% and spots with signal to noise ratio of ≤ 1.25 were excluded from the analysis. Background corrected median intensities of two channels were normalized by using intensity-dependent lowess normalization, and the binding ratio of immunoprecipitated DNA to control DNA was determined for each spot on the microarray [68]. By adopting a single-array error model, a confidence estimate (*p*-value) for each binding ratio was estimated using standard deviations of the two background-intensities [69]. Binding ratios for each of the 2 replicated samples were combined by a weighted average method, and final *p*-values were calculated for the weighted ratios at the 0- and 3-hr time points [69]. A gene was deemed to be bound if its weighted ratio at 3-hr time point is above 2 with *p*-value < 0.01 and showed at least a 30% increase in relative binding compared to 0 hr time point. Then, in order to identify candidate TGF-β/SMAD targets from the bound gene list, genes that showed significant changes in gene expression would be selected after expression microarray analysis.

For expression microarrays, gene expression levels from probe intensities were estimated using a robust multichip average method with quantile normalization and background correction [70]. Principal component analysis (PCA) of 8 Affymetrix arrays showed high variation between the two sets of replicated arrays that were processed on two distinct days (day1 and day2). A two-way mixed model ANOVA analysis with a fixed effect of time variation (0 hr, 3 hr, 6 hr, and 12 hr) and a random effect of day variation (day1 and day2), using Partek software (Partek Discovery Suite 6.2, Partek Inc, St. Louis, MO), was performed to identify and remove the day-to day variation from gene expression estimates. Corrected gene expression estimates, were used for further analysis. Cluster analysis of 8 samples using all the genes, was performed using centered correlation metric and average linkage method. To study the effect of different time points on gene expression, ANOVA analysis was applied. Individual contrasts within the ANOVA model was estimated in order to identify differentially expressed genes that show significant up- or down-regulation at 3, 6 or 12 hr compared to 0 hr using Partek software. Genes with a significant contrast (*p*-value < 0.05) and an absolute fold change over 1.5 were considered as differentially regulated.

Gene promoters that were identified as Smad4-bound at 3 hr (relative to 0 hr) in the ChIP-chip data analysis, and those that showed significantly differential gene expression estimates at 3 hr, 6 hr or 12 hr (relative to 0 hr), were considered to be candidate TGF-β/SMAD targets. Hierarchical cluster analysis of the candidate TGF-β/SMAD target genes was then performed by the centered correlation metric and average linkage method, using expression array data [71].

### Quantitative ChIP-PCR and RT-PCR

To confirm candidate TGF-β/SMAD target promoters, ChIP-PCR was conducted using immunoprecipitated DNAs as templates. Primers (sequences available upon request) were designed to flank a region within 500 bp of the predicted SMAD binding element. Fold-enrichment of amplified DNAs was determined using previously described protocols [42].

Quantitative RT-PCR was performed as described previously [42]. To remove potential DNA contamination, 2 μg of total RNA was treated with DNase I (Invitrogen) and then reverse transcribed with Superscript II reverse transcriptase (Invitrogen). Specific primers for amplification are available on request.

### Ingenuity Pathways Analysis

Ingenuity Pathways Analysis (Ingenuity^® ^Systems, , IPA 6.0) Compare Biomarker feature was used to define common molecules for gene expression (Affymetrix U133A, 1095 molecules) and ChIP-chip data (Agilent Technologies, 2135 molecules) followed by functional analyses aimed to determine the similarities between SMAD-module predicted and IPA-generated targets. The functional analyses run separately on SMAD-predicted and IPA-generated targets identified the biological functions that were most significant to the data sets. Genes from each data set associated with biological functions in the Ingenuity Pathways Knowledge Base (IPKB) were considered for analyses. In addition, IPA Core analysis feature was used to define the signaling pathways common for Affy and SMAD-predicted datasets. More than 800,000 objects currently present in IPKB ontology were used to define algorithmically whether the tested dataset fit into the existing canonical pathways. Fischer's exact test was used to calculate a p-value determining the probability that each biological function assigned to that data set is due to chance alone. Ratios were the relative representation of the number of hits within the tested dataset against the total number of hits within the IPKB.

### Prediction of SMAD and Other Transcription Factor Binding Sites

Sixty-seven SMAD binding elements (SBEs) from human, mouse and rat were collected from the published literature (See Additional File 9 – Table S6). The consensus SBE 5'-CAGAC-3' was extended 6 bp upstream and 4 bp downstream. A SBE position weight matrix on the 15 bp sequences was constructed by formula , where *f*_*b*, *i *_is the number of nucleotide *b *at position *i*, *N *is the number of sequences, and *p(b) *is the background frequency of nucleotide *b*. In the PWM, the 8^th ^and 9^th ^positions were fixed to nucleotide A and G, respectively. The background frequency was determined on 100,000 pseudo-SBE sequences. A 3,000 bp region (-2,000 bp, +1,000 bp), flanking the transcription start site of randomly selected human promoters from the MPromDb database, was extracted [72]. For every AG nucleotide base pair present in the promoter sequence, the flanking regions (-7 bp, +6 bp) were extracted to represent a 15 bp pseudo-SBE sequence. An in-house Perl script was developed that could detect SBEs in Fasta sequences (program available upon request). The core score was the score on the 5 bp consensus SBE; the full score was that on the whole 15 bp sequence. In determining SBEs, the 60-mer sequence on the 44K promoter microarray was equally extended on both directions to a 1,060 bp sequence and scanned for SBE using 0.9 and 0.7 as cutoffs for the core and full scores, respectively. Other transcription factor binding sites that fall within -220 bp to +220 bp of a SBE were of interest in the CART and RF analysis. These binding sites were detected by the MATCH program, using the position weight matrices from TRANSFAC database (TRANSFAC 9.1, minSum profile) [26].

### Random Forest

The Random Forest (RF) classifier is based on growing a large ensemble of classification trees, and the majority vote of the trees determines the class of an observation [21]. Each tree is constructed on a bootstrapped sample from the original data and only a number of randomly selected predictor variables are used in tree branch splitting. This number is a user-defined parameter or set to a default value, i.e. square root of the total number of predictor variables. The result is not sensitive to this number. An estimate of the classification error is supplied by the out-of-bag sample, i.e. the observations that are not used in a particular tree construction. The class label of the out-of-bag sample is predicted by the tree and compared with the true label of the sample. The whole tree ensemble thus generates the misclassification error rate.

While RF is considered as a "black box" method, with no interpretable classification model is present at the end of the application, it still provides useful information, such as variable importance. One of the measures of variable importance is the mean decrease in accuracy, calculated using the out-of-bag sample. The difference between the prediction accuracy on the untouched out-of-bag sample and that on the out-of-bag sample permuted on one predictor variable is averaged over all trees in the forest and normalized by the standard error. This gives the mean decrease in accuracy of that particular predictor variable which has been permuted. Thus, the importance of the predictor variables can be ranked by their mean decrease in accuracy.

For our random forest analysis, we used the RF package in R , with each RF run having 1000 trees in the forest. Stratified sampling with equal sample size in each stratum was employed for the bootstrapped sampling [73]. The dependent variable was the synexpression group labeling derived from the hierarchical clustering of the expression levels of the TGF-β/SMAD target genes. Each predicted transcription factor binding site within the 440 bp sequence, which was centered on a predicted SBE, was considered as a binary predictor variable, with 1 for presence, 0 for absence. Transcription factor binding sites that were present in at least 35% of the sequences of either class were retained in the data matrix for RF, as well as for CART analysis.

### Classification and Regression Tree Analysis

Because of its simplicity and interpretability, CART is one of the most frequently used classification tools [22]. The building of the tree is a 3-stage process. In the first stage, the tree is grown by recursively dividing the data space to binary spaces. The splitting variable and the splitting point can be selected by several criteria. We used the Gini index, defined as , for a node *t *with estimated class probabilities *p(j|t), j *= 1,..., *J*, where *J *is the total number of classes. Once a large tree is grown, the next step is to prune it, until the root node remains in the tree. This pruning procedure, guided by a minimal cost complexity measure, creates a nested subset of trees. The optimum tree is the one with the lowest misclassification error rate by cross validation. CART software (Salford Systems, San Diego, CA) was used in our analysis with equal prior setting and a 10-fold cross validation. The misclassification cost for each class was set to 1. We note that CART differs with RF in that it only builds one tree based on all predictor variables; while RF combines multiple (1000 in our case) trees that are constructed on randomly selected predictor variables.

### Random Forest Variable (TFBSs) Selection for CART Model

When the number of predictors is large and the number of observations is small (so called "*small n large p" *problem in the field of Statistics), CART generally produces a poor classification result due the instability of the individual trees. However, RFs can increase the prediction accuracy as compared to single CART tree, because the ensemble adjusts for the instability of the individual trees induced by small changes in the learning sample, which weakens the prediction accuracy in test samples [22]. Therefore, we first used RF for selecting the most important variables, which were then included in the CART analysis. Because of the randomness inherent to RF, such as random bootstrapping of the data and random selection of the predictor variables for splitting, the most important variables selected by RF would be different from each run. However, the variables that are truly important would consistently appear at the top of the most important list. Therefore, we ran the RF 100 times, with each run having 1000 trees. The variables were then ranked by the average of the 100 runs, with regard to the mean decrease in accuracy.

A series of CART models were then built on the top 30 most important variables in a systematic way. The dataset that the CART model was built upon, initially had only one independent variable (the most important one), and was expanded, by adding more independent variables from the list of the top 30 most important variables, in the order of decreasing importance. The error rates from cross-validation of the CART models are shown in Figure S5A (See Additional File 8). The CART model with the lowest overall misclassification error rate was chosen as the final model. This criterion also agrees with using sensitivity and specificity to judge the model performance (See Additional File 8 – Figure S5B).

The ChIP-chip data are under the accession number [GEO:GSE6727] and the Affymetrix gene expression data are under the accession number [GEO:GSE6653] at the GEO database.

## Abbreviations

TGF-β: transforming growth factor beta; SBE: SMAD Binding Element; TFBS: transcription factor binding site; ChIP-chip: Chromatin Immunoprecipitation (ChIP) followed by microarray analysis; CART: Classification And Regression Tree Analysis; RF: Random Forest algorithm; IOSE: immortalized ovarian surface epithelial cells

## Competing interests

The authors declare that they have no competing interests.

## Authors' contributions

HQ designed the computational methods and performed the statistical analyses. MWYC designed the experimental methods and performed the ChIP-chip experiments. PY coordinated the microarray experiments. SL, DP, IJS, FJA helped with the bioinformatics analyses. CB and ASLC helped with the experimental methods. EVN performed the pathway analyses. HJL, KPN, JHS and LCS participated in the design of the study. RD and TH formulated and directed the design of the study. All authors read and approved the final manuscript.

## Supplementary Material

Additional file 1 **Figure S1. Reproducibility of ChIP-chip experiments**. Normalized log ratios (immonuprecipated DNA over total input DNA) of the biological replicate experiments (0 hrs untreated or 3 hrs TGF-β1-treated) are plotted as smooth scatter plots. Binding ratios for 150 significant genes are indicated by red dots. The overall correlation coefficient of each plot is also shown. Click here for file

Additional file 2 **Figure S2. Reproducibility of expression microarrays**. Dye intensities (log 2) from the technical replicate experiments (0 hrs untreated, 3, 6, 12 hrs TGF-β1-treated) are plotted as scatter plots. Expression data for 150 significant genes are indicated by red dots. The overall correlation coefficient of each plot is also shown. Click here for file

Additional file 3 **Figure S3. Cluster analysis of expression microarray**. Data from expression microarrays were used to perform cluster analysis. The replicates at each time points were technical replicates and were labeled as "Rep1" and "Rep2". The scale bar is "1-correlation". Therefore, the shorter the distance, the stronger the correlation. The result showed that data from the treated and the untreated experiments can be grouped into two different clusters. Click here for file

Additional file 4 **Table S1. List of 150 putative TGF-β/SMAD target genes and their expression levels**. Genes are sorted from most change to lease change according to binding response to treatment. Click here for file

Additional file 5 **Supplementary Tables S2, S3 and S4**. Table S2. Distribution of TGF-β/SMAD target genes. Table S3. Misclassification rates by CART and RF modeling with three synexpression groupsTable S4. Selection of known SMAD co-regulators by RF. Click here for file

Additional file 6 **Table S5. Predicted modules for TGF-β/SMAD target genes**. Column 1 shows the SMAD target gene, column2 gives the predicted SMAD module; columns 3 and 4 show the predicted and observed groups respectively. Click here for file

Additional file 7 **Figure S4. A graphical representation of overlapping molecular and cellular functions in SMAD responsive (from Affymetrix array data) and SMAD target (from ChIP-chip) gene sets from Ingenuity Pathway Analysis**. A graphical representation of overlapping molecular and cellular functions for 73 IPA and 145 SMAD-module predicted targets sorted by a p-value. The significance of each function was calculated by Fischer's exact test (see Methods). Click here for file

Additional file 8 **Figure S5. Choosing the best CART model by step-wise forward variable selection procedure**. **Figure S5A**: Plot of the mean error rates as a function of the number of variables in the CART model (top ranking 30 most important variables selected by RF were used by step-wise forward selection, starting with the most important variable) for dataset 1: up- vs. down-regulated targets and dataset 2: Sustained up- vs. transient up-regulated targets. The error rates were a summation of the error rates of the two classes and were estimated from 10-fold cross-validation. The error rates first dropped and then increased as a function of the number of independent variables. The best CART models, in terms of the lowest overall error rate, consisted of 4 variables for up vs down and 3 variables for sustained up- vs. transient up-regulated targets. **Figure S5B**: The sensitivity versus 1-specificity plot of the CART models. Down regulated target class and transient up-regulated class were selected as positive group for datasets – 1 and 2, respectively. The sensitivity and specificity values were derived from the confusion matrix on the test data reported by the CART software. The point closest to the upper left corner (1-specificity = 0, sensitivity = 1) on each plot was indicated with an arrow, which was the best model in terms of a balance between sensitivity and specificity. For both datasets, equal mis-classification cost rate was used. Consequently, the model with optimal sensitivity and specificity values was also the model with the lowest overall error rate. Click here for file

Additional file 9 **Table S6. List of 67 sequences containing SBEs from the published literature**. Column 1 shows the number of experimentally known binding sites (SBEs) within each target gene; columns 2, 3, 4 and 5 give the gene symbol, Unigene ID, Accession ID and Gene ID respectively; columns 6 and 7 give the relative start and end positions of the SBE (relative to transcription start site); column 8 gives the SBE; columns 9 to 12 give the chromosomal location of the SBE (The genomic coordinates are according to Human NCBI Build 35; Rat Nov. 2004 (rn4) assembly & Mouse NCBI Build 36); columns 13 and 14 give the sequence around SBE (-50 to +50 around the SBE) and its length respectively. Click here for file
