# Supplementary material for: An integrative ChIP-chip and gene expression profiling to model SMAD regulatory modules
Source: BMC Syst Biol. 2009 Jul 17;3:73. doi: 10.1186/1752-0509-3-73 (PMC2724489; doi:10.1186/1752-0509-3-73)
Supplement: Additional file 1 — Figure S1. Reproducibility of ChIP-chip experiments. Normalized log ratios (immonuprecipated DNA over total input DNA) of the biological replicate experiments (0 hrs untreated or 3 hrs TGF-β1-treated) are plotted as smooth scatter plots. Binding ratios for 150 significant genes are indicated by red dots. The overall correlation coefficient of each plot is also shown. [file 1752-0509-3-73-S1.ppt]

## Slide 1
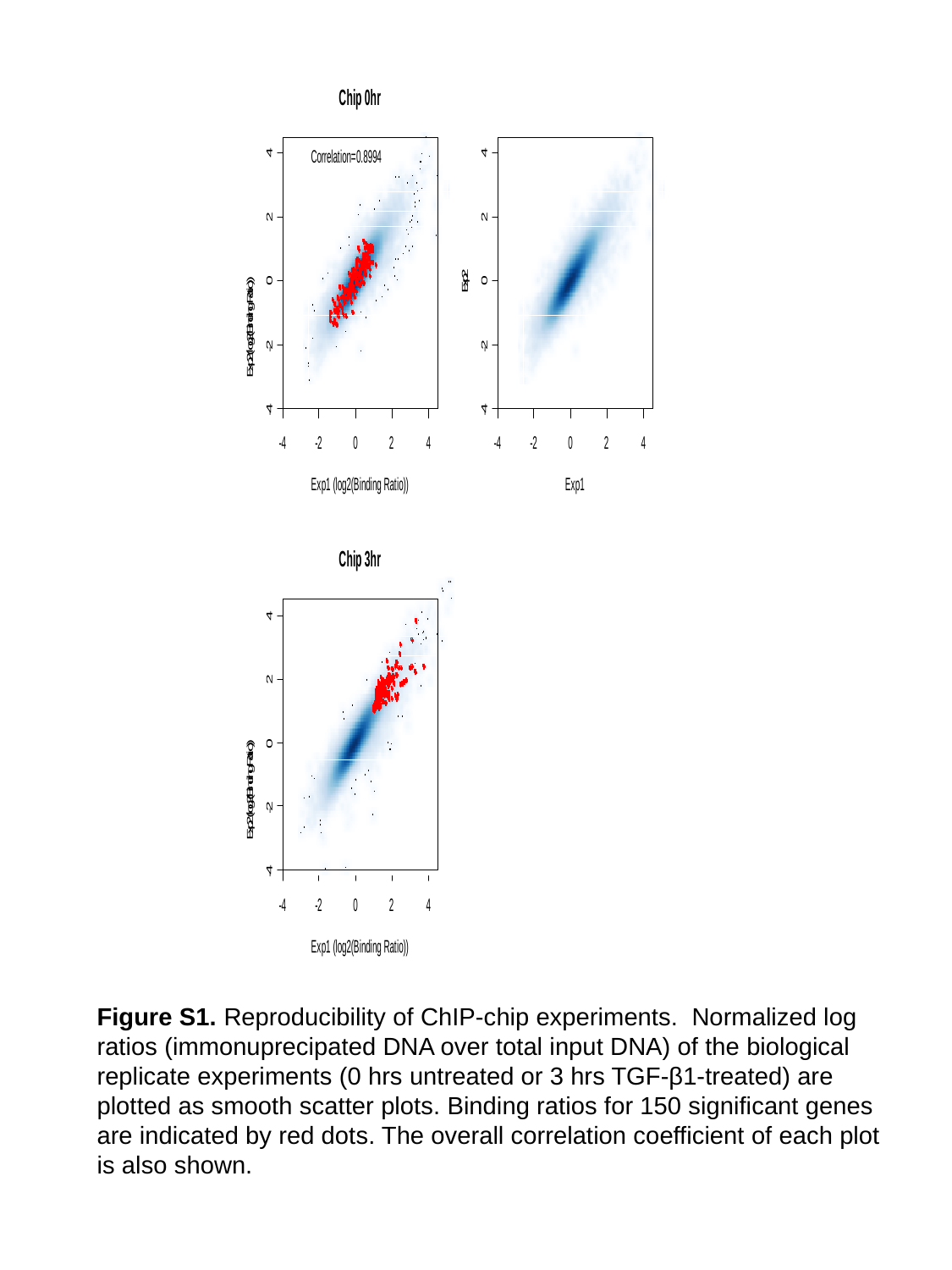

Figure S1. Reproducibility of ChIP-chip experiments. Normalized log ratios (immonuprecipated DNA over total input DNA) of the biological replicate experiments (0 hrs untreated or 3 hrs TGF-β1-treated) are plotted as smooth scatter plots. Binding ratios for 150 significant genes are indicated by red dots. The overall correlation coefficient of each plot is also shown.
