# Supplementary material for: An integrative ChIP-chip and gene expression profiling to model SMAD regulatory modules
Source: BMC Syst Biol. 2009 Jul 17;3:73. doi: 10.1186/1752-0509-3-73 (PMC2724489; doi:10.1186/1752-0509-3-73)
Supplement: Additional file 3 — Figure S3. Cluster analysis of expression microarray. Data from expression microarrays were used to perform cluster analysis. The replicates at each time points were technical replicates and were labeled as "Rep1" and "Rep2". The scale bar is "1-correlation". Therefore, the shorter the distance, the stronger the correlation. The result showed that data from the treated and the untreated experiments can be grouped into two different clusters. [file 1752-0509-3-73-S3.ppt]

## Slide 1
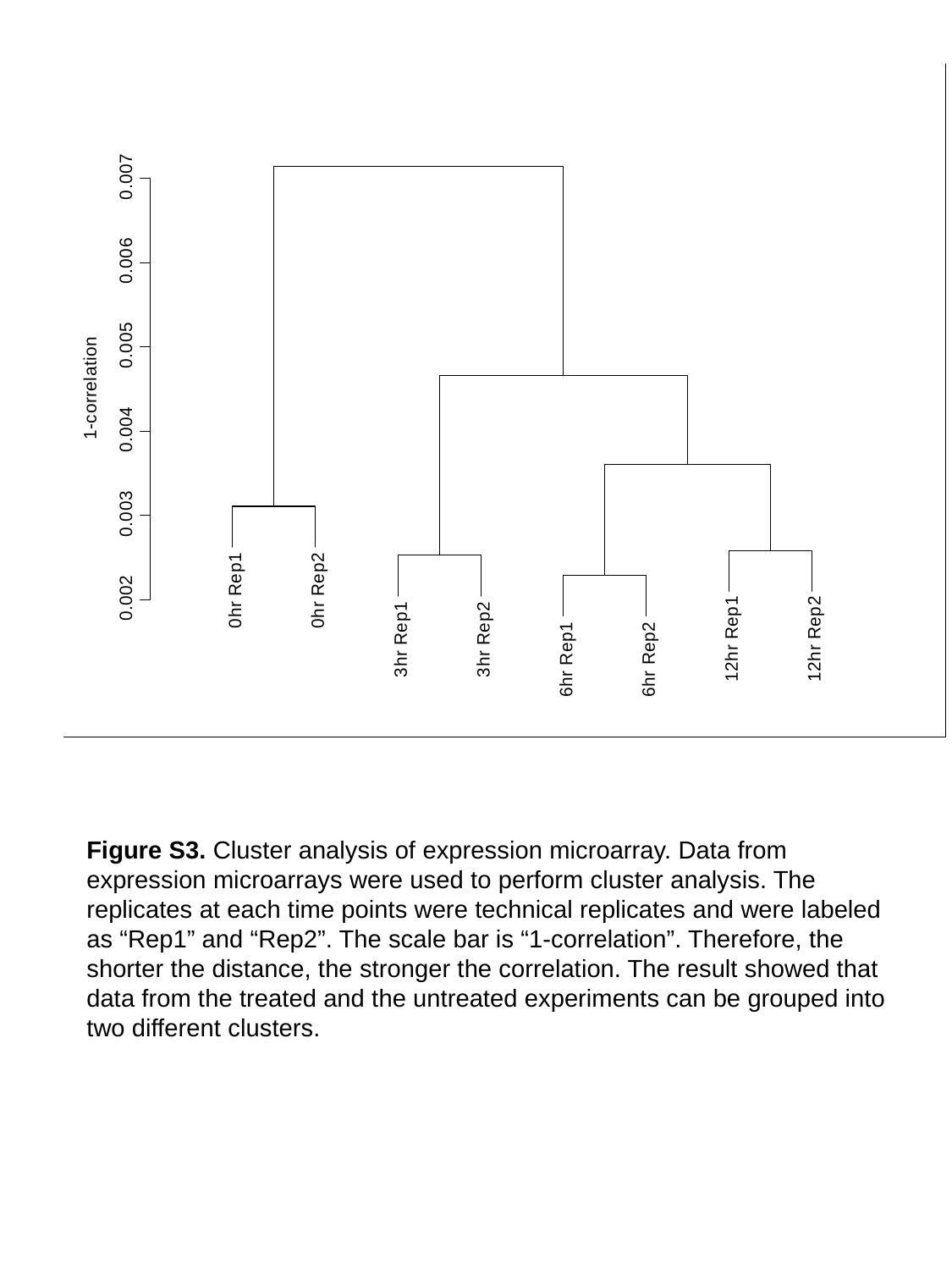

Figure S3. Cluster analysis of expression microarray. Data from expression microarrays were used to perform cluster analysis. The replicates at each time points were technical replicates and were labeled as “Rep1” and “Rep2”. The scale bar is “1-correlation”. Therefore, the shorter the distance, the stronger the correlation. The result showed that data from the treated and the untreated experiments can be grouped into two different clusters.
