# Supplementary material for: An integrative ChIP-chip and gene expression profiling to model SMAD regulatory modules
Source: BMC Syst Biol. 2009 Jul 17;3:73. doi: 10.1186/1752-0509-3-73 (PMC2724489; doi:10.1186/1752-0509-3-73)
Supplement: Additional file 5 — Supplementary Tables S2, S3 and S4. Table S2. Distribution of TGF-β/SMAD target genes. Table S3. Misclassification rates by CART and RF modeling with three synexpression groupsTable S4. Selection of known SMAD co-regulators by RF. [file 1752-0509-3-73-S5.doc]

**Supplementary Tables S2, S3 and S4**

**Table S2.** Distribution of TGF-β/SMAD target genes

| Group | Number of genes | Number of genes  with SBE |
| --- | --- | --- |
| Total | 150 | 124 |
| Group 1 | 1 | 1 |
| Group 2 (Up) | 80 | 65 |
| Top Branch (Sustained) | 54 | 41 |
| Middle Branch (Transient) | 25 | 23 |
| Bottom Branch | 1 | 1 |
| Group 3 | 4 | 4 |
| Group 4 (Down) | 62 | 51 |
| Group 5 | 3 | 3 |

**Table S3.** Misclassification rates by CART and RF modeling with three synexpression groups

|  | Number of  Independent variables | Error rate | | |
| --- | --- | --- | --- | --- |
|  |  | Sustained Up | Transient Up | Down |
| Sample Size |  | 41 | 23 | 51 |
| CART | 170 | 0.88 | 0.66 | 0.49 |
| RF | 170 | 0.64 | 0.93 | 0.52 |
| RF + CART | 6 | 0.49 | 0.26 | 0.57 |

Please refer to the note of Table I.

**Table S4.** Selection of known SMAD co-regulators by RF

| TFBS of Known SMAD co-regulators | Dataset 1  Up vs. Down | Dataset 2  Sustained vs. Transient |
| --- | --- | --- |
| AP-1 | No | No |
| AP-2 | No | No |
| C/EBPβ | No | Yes |
| COUPTF | Yes | No |
| E2F4/5 | Yes | Yes |
| ETS-1 | No | No |
| FoxO | No | Yes |
| GATA4 | No | Yes |
| HIF1α | No | No |
| HNF-4 | Yes | Yes |
| LEF/TCF | Yes | No |
| MITF/TFE3 | No | No |
| MYOCD | No | No |
| NFKappaB | No | No |
| OCT | No | Yes |
| P53 | No | Yes |
| RARγ | Yes | Yes |
| SOX9 | No | No |
| SP1 | Yes | Yes |
| SP3 | No | No |
| STAT3 | No | Yes |
| TCF/β-catenin | No | No |
| Selected | 6 | 10 |
| %selected | 27% | 45% |
