# Supplementary material for: An integrative ChIP-chip and gene expression profiling to model SMAD regulatory modules
Source: BMC Syst Biol. 2009 Jul 17;3:73. doi: 10.1186/1752-0509-3-73 (PMC2724489; doi:10.1186/1752-0509-3-73)
Supplement: Additional file 7 — Figure S4. A graphical representation of overlapping molecular and cellular functions in SMAD responsive (from Affymetrix array data) and SMAD target (from ChIP-chip) gene sets from Ingenuity Pathway Analysis. A graphical representation of overlapping molecular and cellular functions for 73 IPA and 145 SMAD-module predicted targets sorted by a p-value. The significance of each function was calculated by Fischer's exact test (see Methods). [file 1752-0509-3-73-S7.ppt]

## Slide 1
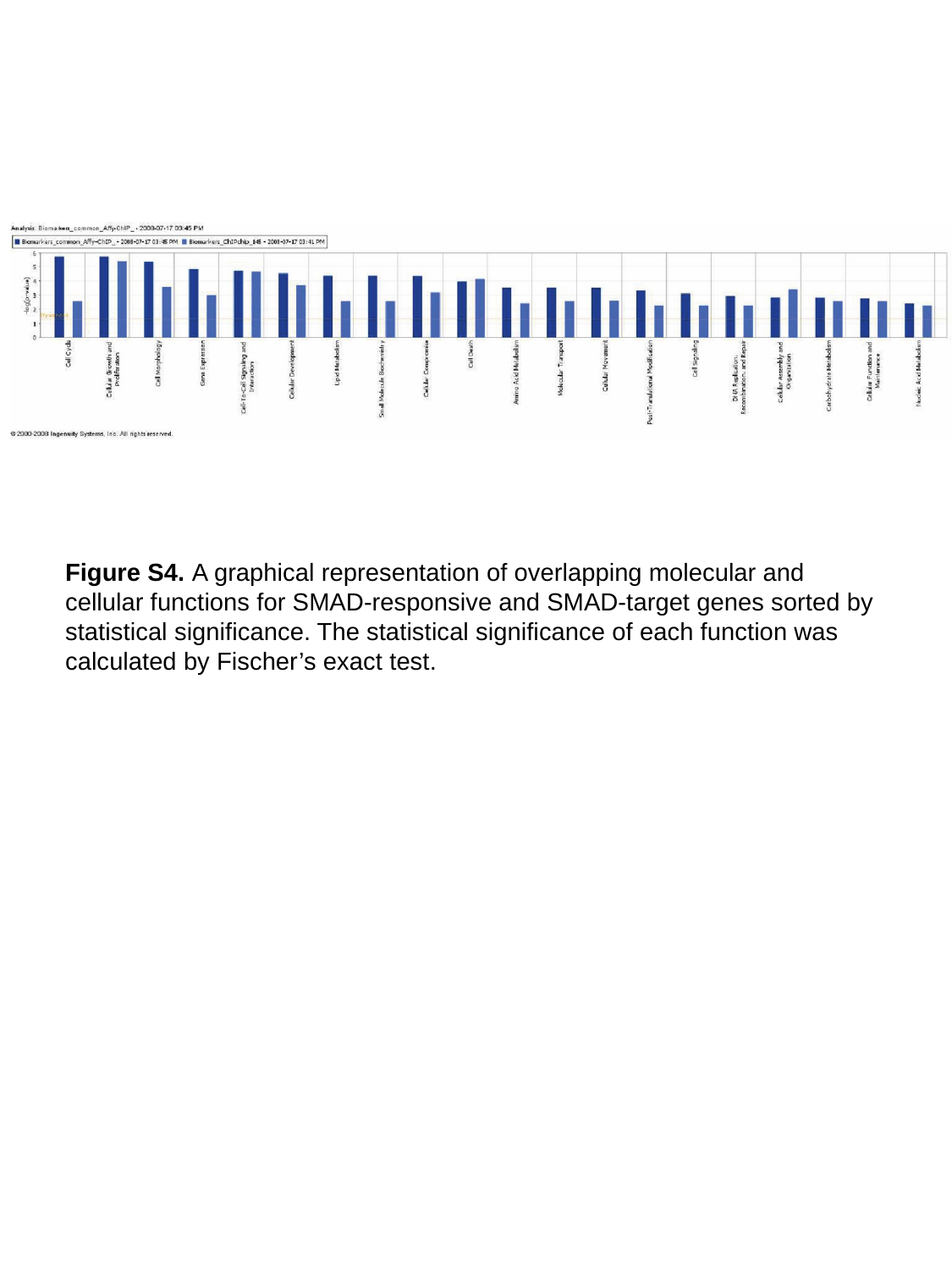

Figure S4. A graphical representation of overlapping molecular and cellular functions for SMAD-responsive and SMAD-target genes sorted by statistical significance. The statistical significance of each function was calculated by Fischer’s exact test.
