# Supplementary material for: An integrative ChIP-chip and gene expression profiling to model SMAD regulatory modules
Source: BMC Syst Biol. 2009 Jul 17;3:73. doi: 10.1186/1752-0509-3-73 (PMC2724489; doi:10.1186/1752-0509-3-73)
Supplement: Additional file 8 — Figure S5. Choosing the best CART model by step-wise forward variable selection procedure. Figure S5A: Plot of the mean error rates as a function of the number of variables in the CART model (top ranking 30 most important variables selected by RF were used by step-wise forward selection, starting with the most important variable) for dataset 1: up- vs. down-regulated targets and dataset 2: Sustained up- vs. transient up-regulated targets. The error rates were a summation of the error rates of the two classes and were estimated from 10-fold cross-validation. The error rates first dropped and then increased as a function of the number of independent variables. The best CART models, in terms of the lowest overall error rate, consisted of 4 variables for up vs down and 3 variables for sustained up- vs. transient up-regulated targets. Figure S5B: The sensitivity versus 1-specificity plot of the CART models. Down regulated target class and transient up-regulated class were selected as positive group for datasets – 1 and 2, respectively. The sensitivity and specificity values were derived from the confusion matrix on the test data reported by the CART software. The point closest to the upper left corner (1-specificity = 0, sensitivity = 1) on each plot was indicated with an arrow, which was the best model in terms of a balance between sensitivity and specificity. For both datasets, equal mis-classification cost rate was used. Consequently, the model with optimal sensitivity and specificity values was also the model with the lowest overall error rate. [file 1752-0509-3-73-S8.ppt]

## Slide 1
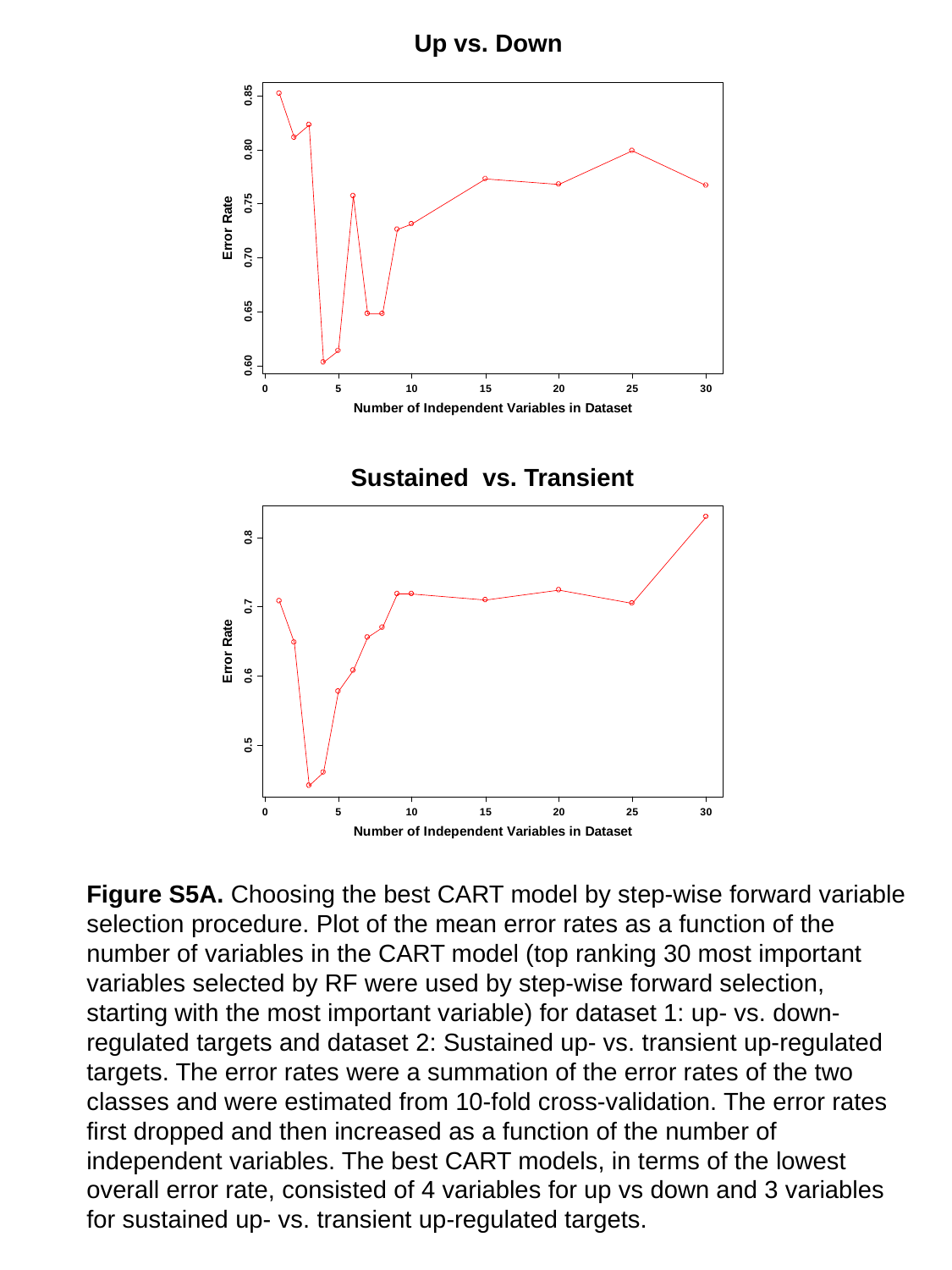

Up vs. Down
Sustained vs. Transient
Figure S5A. Choosing the best CART model by step-wise forward variable selection procedure. Plot of the mean error rates as a function of the number of variables in the CART model (top ranking 30 most important variables selected by RF were used by step-wise forward selection, starting with the most important variable) for dataset 1: up- vs. down-regulated targets and dataset 2: Sustained up- vs. transient up-regulated targets. The error rates were a summation of the error rates of the two classes and were estimated from 10-fold cross-validation. The error rates first dropped and then increased as a function of the number of independent variables. The best CART models, in terms of the lowest overall error rate, consisted of 4 variables for up vs down and 3 variables for sustained up- vs. transient up-regulated targets.

## Slide 2
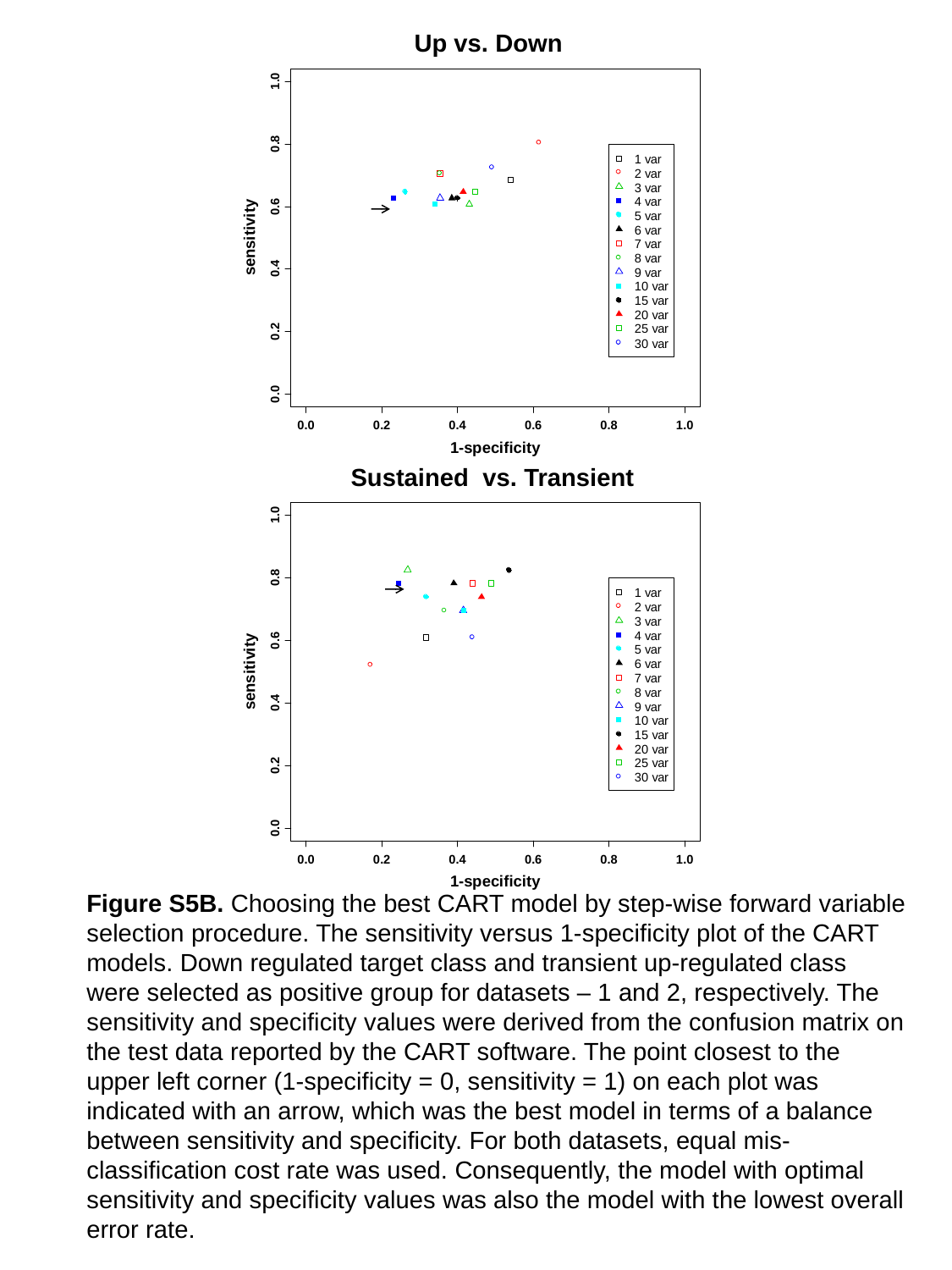

Up vs. Down
Sustained vs. Transient
Figure S5B. Choosing the best CART model by step-wise forward variable selection procedure. The sensitivity versus 1-specificity plot of the CART models. Down regulated target class and transient up-regulated class were selected as positive group for datasets – 1 and 2, respectively. The sensitivity and specificity values were derived from the confusion matrix on the test data reported by the CART software. The point closest to the upper left corner (1-specificity = 0, sensitivity = 1) on each plot was indicated with an arrow, which was the best model in terms of a balance between sensitivity and specificity. For both datasets, equal mis-classification cost rate was used. Consequently, the model with optimal sensitivity and specificity values was also the model with the lowest overall error rate.
